# An ORCID based synchronization framework for a national CRIS ecosystem

**DOI:** 10.12688/f1000research.6499.1

**Published:** 2015-07-06

**Authors:** João Mendes Moreira, Alcino Cunha, Nuno Macedo

**Affiliations:** 1 FCCN, Fundação para a Ciência e Tecnologia, Lisboa, 1700-066, Portugal; 2 HASLab, INESC TEC and Universidade do Minho, Campus de Gualtar, Braga, 4710-057, Portugal

**Keywords:** CRIS, ORCID-CASRAI, PT CRIS, Synchronisation Framework

## Abstract

PTCRIS (Portuguese Current Research Information System) is a program aiming at the creation and sustained development of a national integrated information ecosystem, to support research management according to the best international standards and practices.

This paper reports on the experience of designing and prototyping a synchronization framework for PTCRIS based on ORCID (Open Researcher and Contributor ID). This framework embraces the "input once, re-use often" principle, and will enable a substantial reduction of the research output management burden by allowing automatic information exchange between the various national systems.

The design of the framework followed best practices in rigorous software engineering, namely well-established principles in the research field of consistency management, and relied on formal analysis techniques and tools for its validation and verification.

The notion of consistency between the services was formally specified and discussed with the stakeholders before the technical aspects on how to preserve said consistency were explored. Formal specification languages and automated verification tools were used to analyze the specifications and generate usage scenarios, useful for validation with the stakeholder and essential to certificate compliant services.

## 1 Introduction

PTCRIS ( *Portuguese Current Research Information System*) is a program, officially initiated in May 2014 by FCCN ( *Fundação para a Computação Científica Nacional*), the FCT ( *Fundação para a Ciência e Tecnologia* – the Portuguese Foundation for Science and Technology) unit responsible for planning, management and operation of the national research and education network, a high performance platform for developing and testing advanced communication applications and services. PTCRIS aims to ensure the creation and sustained development of a national integrated information ecosystem, to support research management according to the best international standards and practices.

One of the goals of PTCRIS is to reduce the burden of research output management, by adopting an “input once, re-use often” principle. In order to achieve this goal, a synchronization framework is being developed that relies on ORCID ( http://www.orcid.org/) – a community-based service that aims to provide a registry of unique researcher identifiers and a method of linking research outputs to these identifiers, based on data collected from external sources – as a central hub for information exchange between the various national systems (including CV management systems, open-access repositories, and local CRIS systems) and international systems (WoK, Scopus, Datacite, etc.). Among other features, this framework will enable researchers (or managers) to register a given research output once at one of the interconnected national systems, and have that output automatically propagated to the other ones, thus ensuring global consistency of the stored information. The goal of this paper is to report precisely on the experience of designing and prototyping this synchronization framework.

The design of the synchronization framework followed well-established principles of rigorous software engineering. The main principle is that one should distinguish the *what* from the *how*: in this particular case, *what* is the desired notion of consistency between ORCID and each of the PTCRIS services, and *how* can a synchronization procedure be implemented to enforce such consistency. This allowed us to break down the discussion with the various stakeholders, first seeking an agreement concerning the *what* before dwelling in the technicalities of the *how*.

The second principle is that formal analysis methods and tools should be used to verify that the proposed artifacts follow desirable “well-behavedness” properties. Paraphrasing Richard Feynman, “the first principle is that you must not fool yourself, and you are the easiest person to fool”: the usage of a formal specification language and automatic verification tools allowed us to uncover several corner cases not easy to predict otherwise. In particular, we relied on the formal specification language Alloy ^1^ and its automatic Analyzer. This tool was also used to automatically generate usage scenarios that were useful for requirement elicitation and validation with the stakeholders, but will also be of major importance for certifying compliant PTCRIS services, by allowing rigorous testing of the proposed implementations.

The paper is organized as follows: Section 2 presents a brief overview of (and rationale for) the proposed architecture; Section 3 presents the methodology followed to achieve a trustworthy design for the various components of the framework; Section 4 describes the first prototype that was developed to validate and demo the proposed framework to the community; Section 5 briefly describes some related work; and, finally, Section 6 presents some conclusions and ideas for future work.

## 2 Architecture overview and rationale

**Figure 1.**
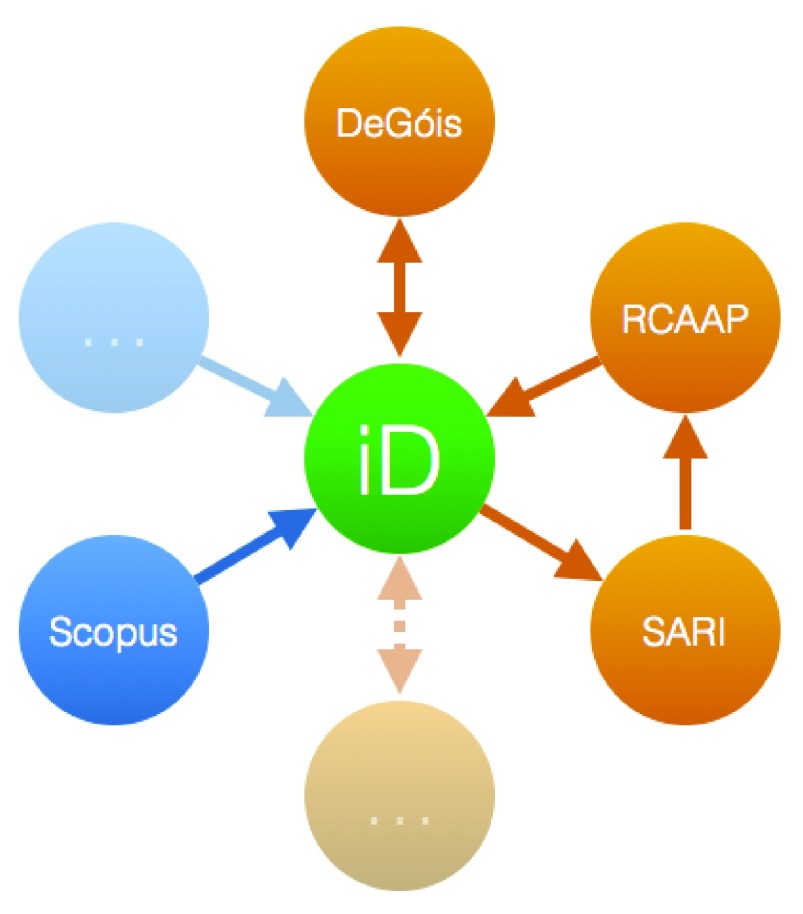
PTCRIS synchronization architecture.

Figure 1 presents an overview of the architecture of the PTCRIS synchronization framework, with some PTCRIS services shown in orange and ORCID sources in blue. PTCRIS is composed of several services with distinct objectives. Among those we have, for example:

**DeGóis** The national academic CV management system ( http://www.degois.pt), where information from researchers is stored and, with the proposed synchronization framework, shared across the PTCRIS ecosystem. DeGóis currently hosts around 22,000 academic CVs.
**RCAAP** The national open-access scientific repository portal ( http://www.rcaap.pt), a platform that acts as an OAI-PMH (Open Archives Initiative Protocol for Metadata Harvesting) aggregator that harvests content from a network of institutional repositories (currently, around 70 in total) and open-access journals. RCAAP currently indexes around 213,000 publications.
**SARI** A DSpace hosting platform for institutional open-access repository services (it currently hosts 26 repositories).

As depicted in Figure 1, not all of these services are expected to synchronize bidirectionally with ORCID. For example, RCAAP will only export research outputs to ORCID, so that they can be harvested by other PTCRIS services. In contrast, institutional repositories (namely those hosted in SARI) will just use ORCID to harvest publications, thus liberating researchers from (the often mandatory task of) having to (manually) insert them. The academic CV management service DeGóis will both import and export research outputs. As the figure also depicts, at least in the earlier stages of deployment of the synchronization framework, some services will still synchronize directly with each other, for example the RCAAP aggregation of open-access publications from institutional repositories will still be performed directly.

There were several reasons that contributed to the choice of ORCID as the central hub for PTCRIS. The main ones are described below:

- High coverage of predefined requirements. The requirements were grouped into three categories: general, functional and technical, and ORCID scored well in all of them. On the general level, items like documentation and support were considered. The main functional requirements were related with both the set of APIs (get, put, etc) and the completeness of the ORCID profile (it also supports most of the CASRAI academic funding CV elements but “services”). Usability issues were also analyzed, as this is a critical issue for PTCRIS. It was concluded that the interface for the integration of systems with ORCID is not only easy to use, but also becoming a standard.
  The technical requirements were related with the easiness of implementation and infrastructure reliability and resilience (ORCID infrastructure is hosted in a world-class datacenter).
- High interoperability with external sources. By the time ORCID was being considered to act as the hub for PTCRIS it was already interoperable with some of the most relevant and important sources (Crossref, Datacite, Scopus and WoK). Furthermore, its interoperability tends to increase as more sources are being added.
- High ORCID coverage of the national research community. In late 2013 – early 2014, due to the research assessment exercise and for the purpose of carrying out a bibliometric study, around 15,000 researchers from Portuguese research units applied for an ORCID iD. These researchers were responsible for more than 90% of the Portuguese scientific output of the 5 years prior to 2013.
- Sustainability. The costs of using ORCID as a hub are very small when compared with the alternative of developing and maintaining a homegrown hub.

Besides the benefits, risks and mitigation measures were also considered when deciding whether to use ORCID as a hub for PTCRIS. The most relevant risk identified was the collapse of the ORCID organization, but the probability of this event was considered to be low. Nevertheless, two mitigation measures were considered: install the hub locally using the ORCID source code (deposited in GitHub); populate the PTCRIS database with the mensal database copy provided to ORCID premium members.

## 3 Specification of the synchronization framework

This section presents the specification methodology that was used to achieve a trustworthy design for the PTCRIS synchronization framework. First, a formal specification of the data models and of the desired consistency predicates was developed. Then, synchronization procedures to enforce such consistency were specified and verified for several “well-behavedness” properties. These formal specifications were also used to automatically derive the already mentioned usage scenarios.

### 3.1 Data model

The synchronization framework operates at the user profile level, that is it intends to synchronize user profiles from the different PTCRIS services with the corresponding user profile from ORCID. The matching of users across these systems is a simple matter, since PTCRIS services can simply store (and most already do) the ORCID iD of the researcher locally. As such, the design of the framework focused only on a single user profile.

The main difficulties in the design of this framework stemmed from fundamental differences between the data model of ORCID and that of most PTCRIS services. As such, before presenting the desired consistency notion and synchronization procedures, we briefly present such data models. Here, we present only a very abstract view of the information stored in such profiles, focusing only on research outputs, namely works, and only on the attributes that are relevant for their synchronization. An ORCID user profile contains additional information, which PTCRIS is also interested in synchronizing among its services. However, some of this information is trivial to synchronize (e.g. education affiliations) while other, albeit not trivial, may be synchronized following the technique presented in this paper for works (e.g. funding information).

**Figure 2.**
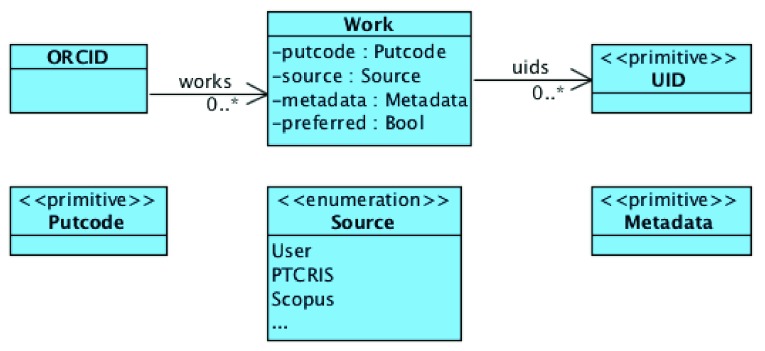
Overview of the ORCID data model.

Figure 2 presents an abstract model of an ORCID user profile. For our purposes, a profile consists essentially of a set of works, each a record containing: a putcode, that uniquely identifies the work internally; a (possibly empty) set of external unique identifiers (UIDs) of the work; the source of the information in the record (which can be the user himself or any other external source associated with ORCID, such as Scopus, CrossRef, or, from now on, a PTCRIS service); any meta-data associated with the work, such as its title, publication year, publication type, authors, etc; and a boolean attribute marking whether the work is the one preferred by the user among *similar* ones (this boolean attribute is not directly returned by the ORCID API, but can be inferred from the order in which the works are stored in an ORCID profile, see discussion below).

A distinctive feature of ORCID is precisely the possibility of using different external sources to automatically populate a user profile. This means that a user profile can contain different works that actually describe the same research output (possibly containing different or even contradictory meta-data). The ORCID web interface already groups together works that describe the same output, showing only the preferred one in the overview. The grouping mechanism is quite simple, and just assumes two works *w*_1_ and *w*_2_ are *similar* if, and only if, they have a shared UID or there is another work *w*_3_ that is similar to both *w*_1_ and *w*_2_. Essentially, this recursive definition considers two works to be similar if, and only if, they share directly or indirectly (via transitivity) some UID.

**Figure 3.**
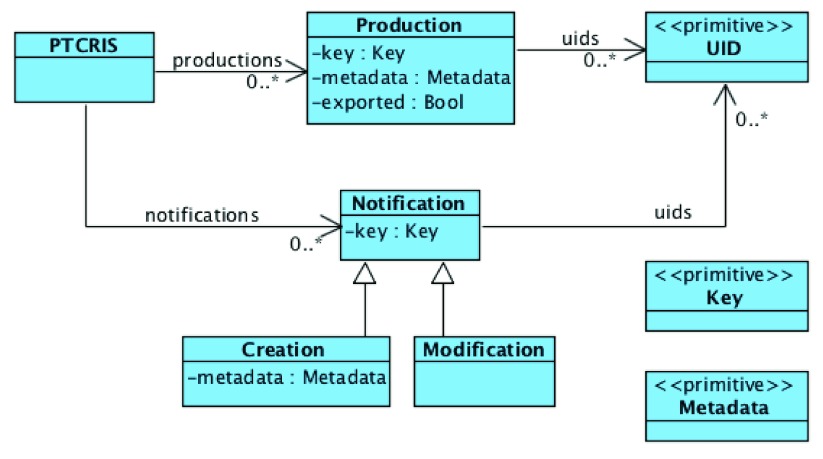
Overview of a PTCRIS service data model.

ORCID imposes several constraints on this data model, such as: there cannot be two works with the same external source with shared UIDs; and among sets of similar works exactly one of them is the preferred one. The ORCID API also forces every work from an external source to have some UIDs assigned, but works added by the user via the web interface may still have an empty set of UIDs. The biggest difference of a user profile in a PTCRIS service (depicted in Figure 3) is that it does not support multiple versions of the same research output, nor the grouping feature of similar versions likewise to ORCID. To avoid confusion with ORCID works we will denote research outputs in PTCRIS as *productions*. The profile of a user in a PTCRIS service is essentially a set of productions, each a record with the following information: a key that uniquely identifies the production; a (possibly empty) set of UIDs; the associated meta-data; and a boolean field indicating whether the production is currently selected be the user to be exported to ORCID.

The PTCRIS synchronization framework is semi-automatic and notification-based. As such, each service will be required to support two kinds of notifications in a user profile: *creation* notifications, to alert the user that a new production has been found in ORCID; and *modification* notifications, to alert the user that new UIDs for an existing production have been found. The latter will be particularly useful for propagating UIDs between different PTCRIS services, in particular from open access repositories that provide *handles* for research outputs to academic CV management services, such as DeGóis.

Likewise to ORCID, this data model is subject to several constraints, such as disallowing exported productions to share UIDs or have no UIDs at all (to comply with the above ORCID guideline).

### 3.2 Consistency predicates

As stated above, we first specified and validated *what* is the desired notion of consistency between ORCID and each of the PTCRIS services. Formally, this consistency is a predicate of type ORCID × PTCRIS → Bool, that given two user profiles returns a boolean indicating whether they are consistent with each other. Typically, this consistency predicate is specified as a set of logical rules that must all be satisfied to render the profiles consistent.

The consistency between ORCID and a PTCRIS service was factorized in two modular consistency predicates whose rules were precisely defined in the design phase:

IMPORTED : ORCID × PTCRIS → Bool This consistency predicate should be enforced by every PTCRIS service that wishes to rely on the synchronization framework to harvest research outputs from ORCID, namely new publications and new UIDs of known publications. The general principle of IMPORTED is that every UID in ORCID should be harvested. The enforcement of this consistency predicate should be semi-automatic, based on a notification system, giving freedom to the user to select which outputs or UIDs he wishes to add to his PTCRIS profile.
EXPORTED : ORCID × PTCRIS → Bool This consistency predicate should be enforced by every PTCRIS service that wishes to be an ORCID source, and export its productions to ORCID, ensuring that other PTCRIS services can harvest them. The general principle of EXPORTED is that every exported production should be stored as a work in ORCID and then automatically kept up-to-date.

These consistency predicates are logically independent, in the sense that each can either hold or not, independently of the value of the other. A PTCRIS service may also wish to implement the conjunction of both, leading to a consistency predicate we denote as (fully) SYNCED:

SYNCED : ORCID × PTCRIS → Bool

SYNCED( *o*, *p*) $\dot{=}$ IMPORTED( *o*, *p*) ∧ EXPORTED( *o*, *p*)

Since the PTCRIS services do not support grouping likewise to ORCID, some caution must be exercised to avoid the proliferation of productions and notifications that describe the same research output. In particular, when an ORCID work is unknown to the PTCRIS service, the existence of a single creation notification, grouping all UIDs of its similar works should suffice to ensure consistency. This is just one of the rules that must be satisfied for IMPORTED to hold. IMPORTED is mainly focused on UID harvesting, the consistency of the meta-data being a secondary concern. However, meta-data still needs to be filled in when a creation is notified following the discovery of a group of (unknown) similar works. Since their meta-data can (and often does) differ, it is not clear how this meta-data extraction should be performed. On first glance, the obvious choice would be to pick the meta-data of the preferred work. Unfortunately, the following reasons prevent us from currently enforcing this behavior:

- Since all groups of similar works must have a preferred work (essentially the one chosen to be displayed in the user web page), a default preferred is always chosen by ORCID when a new research output is imported or the current preferred one is deleted by the user.
- The ORCID API does not currently distinguish such default preferred works from user-selected ones.
- This means that the user might not have sanctioned the meta-data present in his preferred works at the time they are being imported into a PTCRIS service.
- Unfortunately, meta-data is of highly variable quality in ORCID, with some sources currently publishing meta-data with gross mistakes, for example, wrong publication types.

As such, the choice of how to fill in the meta-data in creations was currently left for each PTCRIS service. Some will ignore the preferred and just allow the user to rank sources according to the perceived quality of their metadata, and then try to choose the meta-data of the work from the highest ranked source.

The EXPORTED consistency predicate is considerably simpler than IMPORTED. Essentially, the specified consistency rules force that there must exist a one-to-one correspondence between exported productions and works in ORCID whose source is the PTCRIS service.

### 3.3 Synchronization procedures

When the user profiles at ORCID and at the PTCRIS service are inconsistent *how* can they be automatically synchronized to recover the consistency? To achieve that, we have specified two separate synchronization procedures to be used when the service intends to enforce consistency according to IMPORTED or EXPORTED, respectively. These modular synchronization procedures can also be combined in a precise way, to recover the consistency in services that are enforcing both consistency predicates.

IMPORT : ORCID × PTCRIS → PTCRIS This synchronization procedure should be used to enforce the IMPORTED consistency predicate. The main principle is that it does not change the user profile in ORCID. Moreover the only changes it produces to the PTCRIS profile is to add and remove notifications.
EXPORT : ORCID × PTCRIS → ORCID This synchronization procedure should be used to enforce the EXPORTED consistency predicate. The main principle is that it does not change the user profile in PTCRIS. Moreover the only changes it produces to the ORCID profile is to add / delete / modify works whose source is the PTCRIS service.

With the help of automatic formal verification tools, the specified synchronization procedures were checked for several “well-behavedness” properties. The most important of those is *correctness*, that ensures that after running the synchronization procedures the user profiles in ORCID and in the PTCRIS service are indeed consistent:

IMPORTED( *o*, IMPORT( *o*, *p*))

EXPORTED( EXPORT( *o*, *p*), *p*)

Another important “well-behavedness” property is *stability*, ensuring that if we run the synchronization procedures on already consistent states the result is the same (modulo differences in keys):

IMPORTED( *o*, *p*) ⇒ IMPORT( *o*, *p*) = *p*

EXPORTED( *o*, *p*) ⇒ EXPORT( *o*, *p*) = *o*

Having *stable* synchronization procedures ensures that there is no need to explicitly check the consistency to determine if they should be run. If both user profiles are consistent, running the specified procedures would not affect them. In fact, the checking procedures have the same approximate complexity as the synchronizing procedures, and thus, no significant performance gains would be achieved by running them beforehand.

The two specified synchronization procedures can be combined to obtain a synchronization procedure that enforces SYNCED, the full consistency of the user profiles according to both IMPORTED and EXPORTED (to be used by services that wish to enforce both):

SYNC : ORCID × PTCRIS → ORCID × PTCRIS

SYNC( *o*, *p*) $\dot{=}$ let *o*′ = EXPORT( *o*, *p*)

in ( *o*′, IMPORT( *o*′, *p*))

The specified order of execution is not arbitrary. In fact, it is the only order that ensures that the resulting procedure is both *correct* and *stable*:

SYNCED( SYNC( *o*, *p*))

SYNCED( *o*, *p*) ⇒ SYNC( *o*, *p*) = ( *o*, *p*)

In particular, if the user profiles in ORCID and PTCRIS are not consistent according to EXPORTED, running the EXPORT procedure can make them inconsistent according to IMPORTED. As such, IMPORT must be run after EXPORT to ensure that full consistency is attained. A concrete example is presented in the following section.

### 3.4 Usage scenarios

The formal specification of the data models, consistency predicates, and synchronization procedures, allowed the usage of automatic analysis tools (namely, the Alloy Analyzer model finder) to generate a large number of diverse usage scenarios. This section presents one of the generated scenarios. Although small, we believe it is interesting enough to convey the usefulness of this process for requirement validation and for implementation testing.

**Figure 4.**
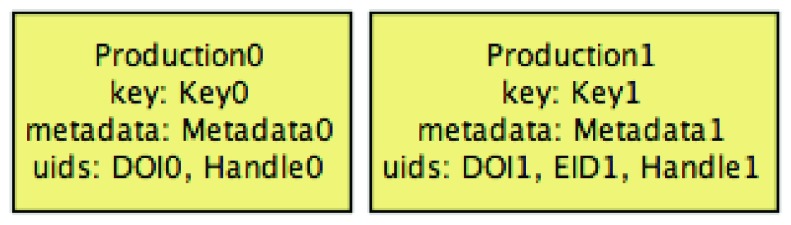
Initial PTCRIS profile.

As the initial state of this scenario, consider the PTCRIS and ORCID user profiles presented below, over which both the IMPORTED and EXPORTED consistency predicates are enforced (simulating, for instance, the DeGóis CV management system). The PTCRIS profile consists of two productions, none selected to be exported, that do not share UIDs, and thus cannot be considered similar, as depicted in Figure 4.

**Figure 5.**
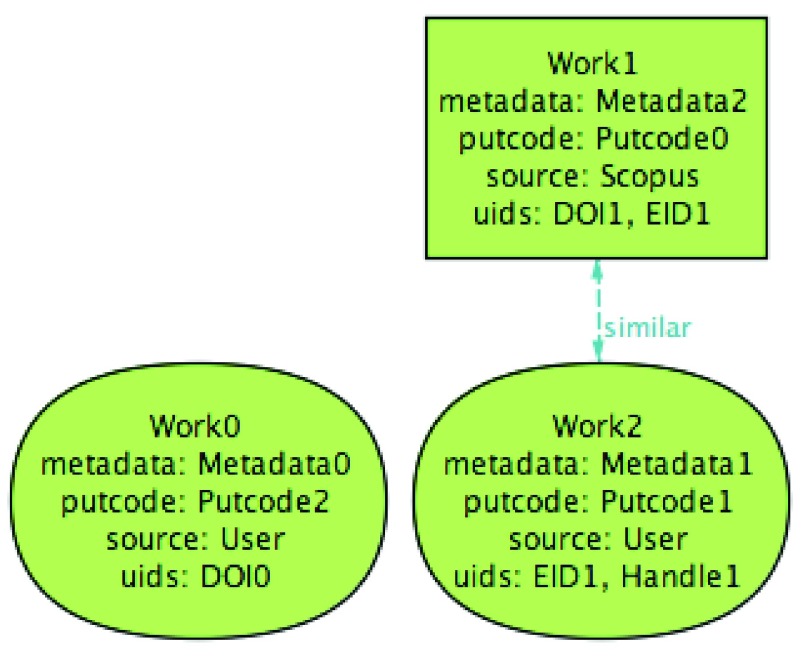
Initial ORCID profile.

The ORCID profile contains two groups of similar works that correspond to the two productions, depicted in Figure 5 (preferred works are depicted with round shapes).

Even though the UIDs of Work1 and Work2 are not exact matches (nor their meta-data), they both share the EID1 identifier, and thus are considered similar and grouped by ORCID. These two profiles are IMPORTED-consistent because all UIDs from ORCID are known to the PTCRIS: Production0 contains the identifiers from Work0 while Production1 aggregates the identifiers from Work1 and Work2. Note that the PTCRIS productions actually contain additional UIDs not known to ORCID; this does not affect the consistency of the profiles since the goal of IMPORTED is to harvest information from ORCID to the PTCRIS service. Since no production is selected to be exported, the profiles are also EXPORTED-consistent.

**Figure 6.**
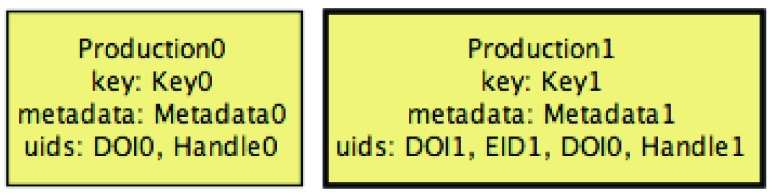
PTCRIS profile after user update.

Now imagine that the user, after examining the production’s meta-data concluded that the two productions at the PTCRIS profile actually represent the same research output. To unify them, the user introduces a UID from Production0 in Production1 (e.g., DOI0), rendering them similar. Then, this update can be propagated to other services by exporting Production1 to ORCID that acts as the research hub, as depicted in Figure 6 (productions set to be exported are denoted by bold frames).

**Figure 7.**
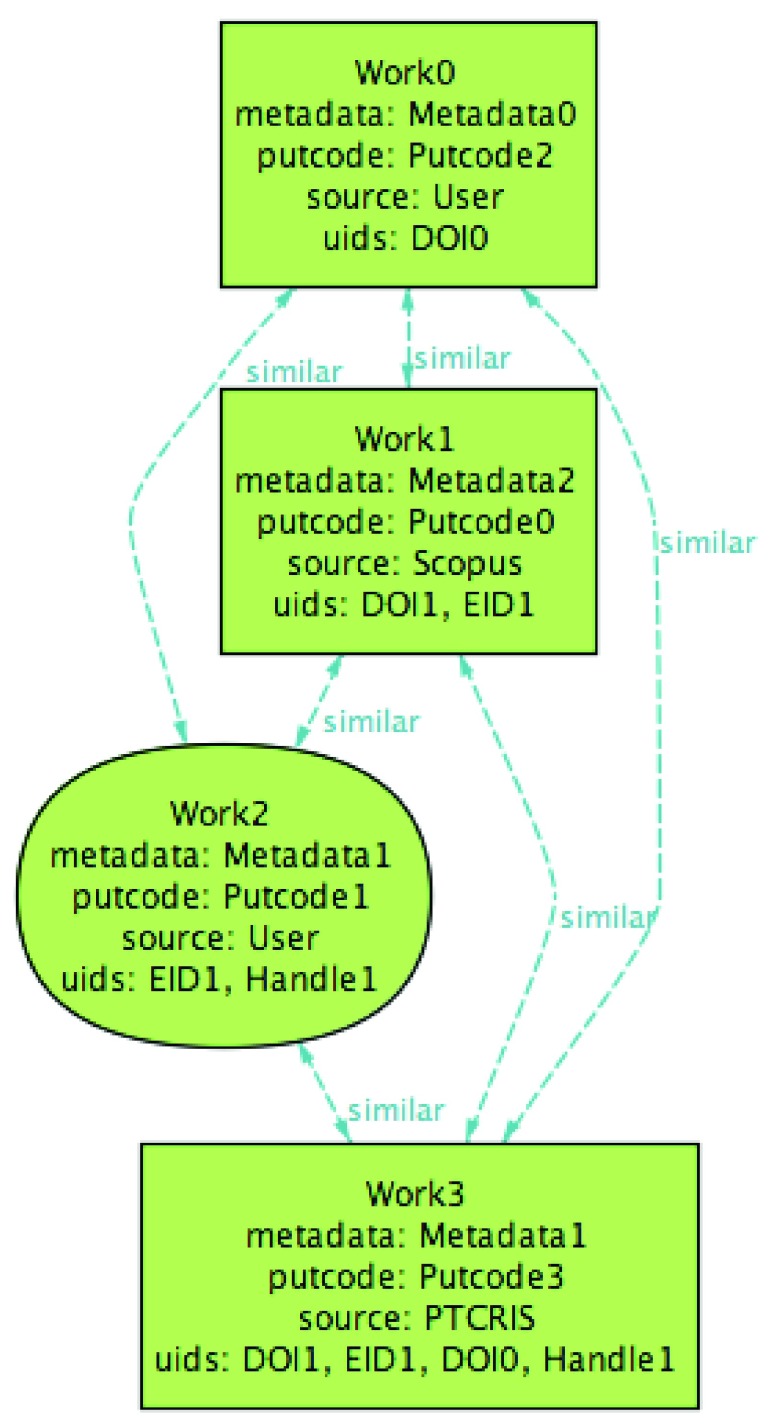
ORCID profile after EXPORT execution.

At this point, the profiles are no longer EXPORTED-consistent, so an identical ORCID work must be created from the exported production. After running the EXPORT procedure, the updated ORCID profile is depicted in Figure 7.

**Figure 8.**
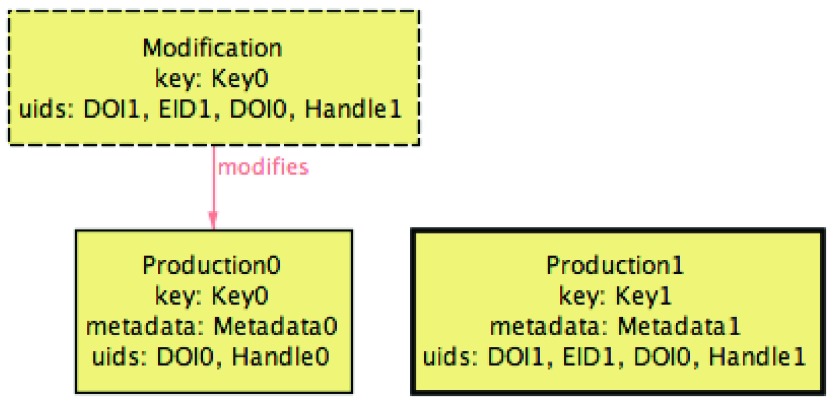
PTCRIS profile after IMPORT execution.

This update reflected the intentions of the user: the introduction of Work3 in the ORCID profile due to the exportation of Production1, unified the two groups under a single group of similar works. (In this scenario, Work2, one of the preferred works in the initial ORCID profile, was preserved as the preferred, while Work0 was demoted. At the moment it is not clear how ORCID would select the preferred in this situation, so our specification also considers as a acceptable possible outcome a profile where Work0 is the preferred one.) However, this has consequences to the IMPORTED-consistency of the profile, since productions related with Work0 need now be updated with the new UIDs. Thus, this update needs to be propagated to all other relevant services (like the SARI repositories), but also to the PTCRIS service that triggered this update, since it was assumed to enforce both IMPORTED and EXPORTED Concretely, when IMPORT is run back to the PTCRIS profile, Production0 is matched with the whole group of works, resulting in the profile depicted at Figure 8, where a modification notification is associated to Production0 to add all harvested UIDs.

Since the EXPORT procedure may introduce IMPORTED-inconsistencies, the service enforcing both consistency predicates should always run IMPORT after EXPORT, that is, the SYNC procedure specified above.

## 4 Prototype implementation

As proof of concept, a prototype of this synchronization framework was implemented and shown to the FCCN community in its annual meeting, held in February 2015 ( http://jornadas.fccn.pt). In this prototype, the following PTCRIS services and systems were involved:

**DeGóis** The national CV system, already supporting ORCID iDs and a preliminary version of the IMPORT and EXPORT synchronization procedures. This new version of this system is expected to be released to the community in the 3rd Quarter of 2015.
**RCAAP** The national OAI-PMH aggregator, implementing a preliminary version of the EXPORT synchronization procedure.
**SARI** The DSpace platform used to provide institutional repository services. Version 5.1 of DSpace was used with some minor adjustments aimed to support ORCID iD (for the purpose of the demo only). Notice that the current version of DSpace, branch JSPUI, still does not support ORCID.
**OJS** Platform used to provide hosting to open-access journals. Like DSpace, the *Open Journal System* was used with minor adjustments to support ORCID iD.

The demo at the aforementioned event involved the following steps:

1. At DeGóis, create a new user profile and run IMPORT to populate it with research outputs harvested from ORCID.
2. Insert a new production at DeGóis (entitled “ *The gap between technologies and science*”) and run EXPORT to send it to ORCID.
3. At OJS submit and approve a new article from the same researcher (entitled “ *Registo submetido a Revista com OJS 1*”).
4. At SARI deposit an article (entitled “ *Portuguese repositories bloom : the RCAAP project*”) in a institutional open-access repository.
5. These two articles are harvested by RCAAP, and then the researcher runs EXPORT to send them to ORCID. The state of the user profile at ORCID after these steps can be seen in Figure 9.
  **Figure 9.**
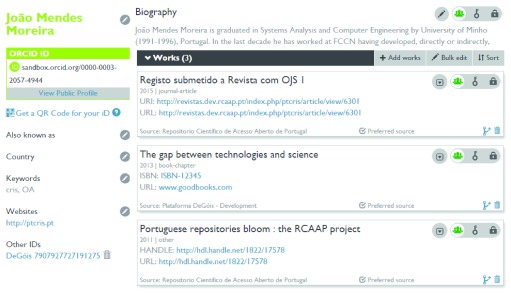
User profile at ORCID.
6. At DeGóis run the IMPORT procedure to harvest these two outputs. The state of the user profile at DeGóis after this step can be seen in Figure 10.
  **Figure 10.**
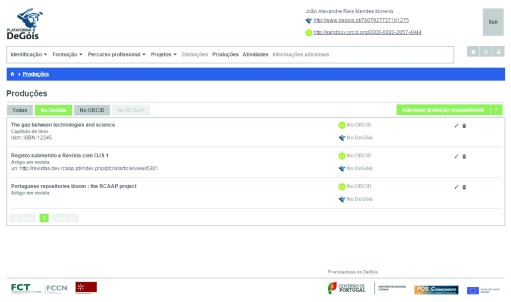
User profile at DeGóis.

This prototype showed, in our opinion, compelling and real use cases for the synchronization framework. The prototype still differs from the upcoming release version of the framework in the following aspects:

- The RCAAP portal still does not implement the final version of the EXPORT procedure, that relies on ORCID Metadata Round Trip functionality to automatically feed updates to the ORCID profile.
- The DSpace based SARI platform does not yet implement the IMPORT procedure. From the end user perspective, this is one of the most expected features, since it will free researchers from manually filling in meta-data. From the FCT perspective, it is also of critical importance, as the notification based synchronization service will increase the output deposit rate, and thus facilitate its open-access mandate.

## 5 Related work

The laws presented in Section 3.3 are standard “well-behavedness” laws in synchronization frameworks, namely on those for bidirectional transformation, whose goal is precisely to maintain two artifacts consistent by means of two transformations that propagate updates from each to the other (for an overview of this research field please see 2). To be more precise, our formalization is based on the concrete framework of constraint maintainers, proposed by Meertens ^3^, and later used by Stevens ^4^ to formalize the OMG (Object Management Group) standard bidirectional transformation language QVT-R (Query/View/Transformation - Relations) ^5^. In fact, an interesting question is whether the domain specific QVT-R language could be used instead of the general purpose Alloy to formalize the consistency predicates, and later used with a QVT-R engine (for example, the Echo tool ^6, 7^) to implement the synchronization procedures.

Alloy and its Analyzer have been previously used in the validation of transformation specifications, namely for transformations specified in QVT-R ^8^ and ATL (ATLAS Transformation Language) ^9^. Likewise to these approaches, we have also used Alloy to verify properties of the specified consistency predicates. However, we also relied on its model finding functionalities to generate scenarios that helped the different stakeholders consensually establishing the system’s requirements.

CV management systems and open-access repositories typically connect with ORCID only in the IMPORT context and only support creation notifications, not allowing the user to EXPORT research outputs back to ORCID. Such is the case of services like Impactstory ( http://impactstory.org), ScienceOpen ( http://www.scienceopen.com) and Symplectic’s Elements ( http://symplectic.co.uk/products/elements). The exception is Thomson Reuters’ ResearcherID ( http://www.researcherid.com), which also aims to provide a unique researcher identifier, and that allows the user to export research outputs back to the ORCID profile. Interestingly, some of these services, like Impactstory and Elements, resort to ORCID only to harvest UIDs and then retrieve meta-data from other trusted services, ignoring the actual ORCID works. As a consequence, any grouping of works (possibly enforced by the user) is ignored, contradicting the perspective of ORCID as a central hub for research outputs (in our scenario, there would be a different notification for each supported UID). For ORCID works without UIDs assigned, Impactstory retrieves the ORCID meta-data, while Elements currently ignores such works. In contrast, ResearcherID considers each ORCID work as an independent entry, not embracing its essence as an aggregator of research outputs from varied sources, which may lead to several duplicated entries (in our scenario, there would be a different notification for each work). Our proposed approach sits between these two approaches: while IMPORT does focus on the retrieval of UIDs, it also considers how these UIDs are grouped in the ORCID profile. In the EXPORT context, ResearcherID, unlike our approach, does not keep track of previously exported outputs. Since the ORCID API does not allow sources to introduce works with repeated UIDs, the user is currently not able to update works from ResearcherID without previously deleting them from the ORCID profile. Outputs without UIDs are duplicated in the ORCID profile when exported; our framework forbids the exportation of productions without UIDs to avoid this issue.

## 6 Conclusion

This paper reported on the experience of developing an ORCID based synchronization framework for PTCRIS. This synchronization framework was recently prototyped and demoed at a national research community event, receiving quite positive feedback. During its design, formal analysis techniques and tools were used with excellent results, in particular to automatically generate usage scenarios (namely, corner cases) that proved very useful to help clarify and validate the requirements with the stakeholders. The first stable and detailed specification of the synchronization framework will be made available soon as an open-access report. We expect to have certified implementations of that specification in DeGóis, RCAAP, SARI, and two local CRIS systems by the end of 2015.

This paper is based on the ORCID API v1.2. Version 2.0, currently in early stages of development, is expected to affect some details of the described synchronization framework, but not the overall concepts. The most relevant change is that groups of similar works, as well as the preferred among them, will become explicit in the ORCID data model. This will simplify the IMPORT procedure, since it will no longer need to compute the groups of similar works.

In a future version we intend to design alternative IMPORT and EXPORT procedures with more sophisticated behaviors. For example, depending on user feedback we may consider implementing a notification dismiss feature, to accommodate users that may want to register different research outputs in their ORCID and PTCRIS profiles. Another possible interesting feature would be to allow IMPORT to somehow recognize edits to works in ORCID and automatically incorporate them in the respective PTCRIS profile.
