# Supplementary material for: An ORCID based synchronization framework for a national CRIS ecosystem
Source: F1000Res. 2015 Jul 6;4:181. [Version 1] doi: 10.12688/f1000research.6499.1 (PMC4536610; doi:10.12688/f1000research.6499.1)
Supplement: Peer review history

# Peer review: Referee response for version 1

**DOI:** 10.5256/f1000research.6975.r9344

**Published:** 2015-08-07

**Authors:** Simeon Warner

**Affiliations:** 1 Division of Library Information Systems, Cornell University Library, Cornell University, Ithaca, NY, USA

This article describes work to use ORCID iDs as the basis for synchronization of data between distributed components of a national CRIS system in Portugal. Portugal has very high ORCID coverage because of the mandated use of ORCID iDs for a 2013/2014 research assessment exercise, and thus provides a good test-bed for ORCID iD based synchronization. The background for this work and the architecture used are well described in the introductory sections.

My principal criticism of the article is that it describes a formal modeling process, that was used to assess and refine the synchronization strategy, in insufficient detail for a reader assess the correctness of or repeat the analysis. Yet, the description of this modeling process nonetheless introduces a number of formalisms that distract from the flow of the article. Ideally, the description of the formal modeling process would be separated out and then expanded to a point where, along with the Alloy source files, the reader could repeat the analysis (plans to provide these details are mentioned in the conclusion, the article should be updated to link to them when available). Because this is a descriptive software tools article, I feel that this deficiency does not undermine the article's usefulness or conclusions.

The later sections of the article describe the prototype implementation, a modest amount of additional work to be completed before a production release, and related work. A number of other issues would benefit from discussion, such as the handling of edits to works in ORCID and other sources; user testing, understanding and acceptance of the system; and limitations on the type of research output included beyond publications.

Overall I feel this is a useful and timely description of new work in an evolving area and thus approve it as is. I hope the authors will follow-up with an article evaluating the system in production.

I have read this submission. I believe that I have an appropriate level of expertise to confirm that it is of an acceptable scientific standard.

# Peer review: Referee response for version 1

**DOI:** 10.5256/f1000research.6975.r9347

**Published:** 2015-07-28

**Authors:** Keith G. Jeffery

**Affiliations:** 1 Keith G. Jeffery Consultants, Faringdon, UK

The paper describes a synchronisation method across multiple research information systems Holding CV information and full-text articles with metadata) in Portugal using ORCID as the essential linking / identification mechanism.  The synchronisation protocol is specified formally and a prototype has been constructed which is reported to have been received successfully.

The paper provides a useful advance in attempts to ensure consistency in  research output publications and thus should be made widely available.

There are several aspects of relevance which are not covered - or covered poorly - in the paper.

1. The paper does not 'stand back' and describe that the need for synchronisation arises because of a lack of policy by national research funding organisations in terms of what should be recorded/stored and how it should be recorded/stored. This policy vacuum has led - in all countries not just Portugal - to the provision of multiple independent incompatible systems and inconsistent information;
2. The synchronisation discussed covers only publications; there are many other aspects of research information that may be recorded inconsistently in multiple systems. This restriction  should be made clear;
3. The data models do not seem to be recording temporal information about (a) the valid time (interval) relating to the publication and (b) the transaction time relating to the publication appearance and any subsequent changes in the digital stored representation. These temporal elements are necessary for precedence, provenance and version differentiation. The need for and lack of temporal information should be discussed;
4. The mechanisms for grouping 'like' publications are likely to produce inconsistent results.  Relying one one or more equivalent identifiers is clearly not sufficient and the paper correctly indicates that metadata may be inconsistent. However, some measure of 'separation distance' based on metadata element values could - and probably should - be used. This would be facilitated by point 5 below;
5. The data models used in the participating systems are very simple and do not really reflect the real world. There exist in the real world complex inter-relationships between (versions of) publications (and indeed between publications and other entities in the research world such as persons, organisations, projects, funding, datasets, software, equipment...) and their correct representation requires a syntax and semantics beyond simple identification. The use of an overarching, superset data model in PCTRIS with formal syntax and declared semantics (thus ensuring referential and functional integrity) and providing a superset over the metadata of the collection of connected systems would give a greater chance of correct assignment of publications to groupings of 'like' publications;

However, as indicated, the paper provides a useful advance in the state of the art and forms a good basis for discussion of more complete solutions to a very difficult problem.

I have read this submission. I believe that I have an appropriate level of expertise to confirm that it is of an acceptable scientific standard, however I have significant reservations, as outlined above.

# Peer review: Referee response for version 1

**DOI:** 10.5256/f1000research.6975.r9346

**Published:** 2015-07-27

**Authors:** Mikael K. Elbaek

**Affiliations:** 1 Technical Information Center of Denmark, Technical University of Denmark, Kongens Lyngby, Denmark

The paper by Moreira *et al.* provides an interesting and relevant proto-type solution to the synchronization problem of several parallel research information systems in a national CRIS (Current Research Information Systems) setting. In the Portuguese CRIS landscape there are no local CRIS-systems (i.e. Pure, Converis, Symplectic etc.) in order to understand the Portuguese implementation it is important to make this distinction that the PTCRIS is an umbrella (or agregator) that collects data from several sources including a network of repositories SARI (DSpace), a national research portal (RCAAP) an OAI-PMH service provider, e.g. aggregator and a national CV management system (DeGóis). PTCRIS in that sense is not one system or piece of software. Even though this architecture may be unique to Portugal the implications of using ORCID as a syncornization framework is interesting and relevant for other national Research Information/CRIS-architectures. The result is interesting and important for developers of national and international research infrastructures – this is why there are also a few points where the paper may be improved.

1. In the paper the authors provides an analytic and generic approach to specifying the relevant synchronization procedures asking first *what* and then *how. *The article does not go into the details of the *what *but describes briefly how this data has been collected and analysed. In this reviewers opinion a more detailed description of the *what *and a description of the selected use-cases would provide a more comprehensible analysis and clear definition of the selected scenarios. Currently the authors provides abstract descriptions of the synchronization scenarios (see chapter 3.2: Consistency predicates). Because of this level of abstraction it is not completely clear who the end-users are, is it the system managers, system owners, research managers or the researchers (authors and owners of the ORCID profiles).
2. The proposed framework is focussing on the technical implementation of synchronization. But how will the users or researchers e.g. the ORCID profile owners influence the robustness of the synchronization job. One example is that ORCID profile owners may not want to have the same records (works/productions) in their PTCRIS profile as the want in the ORCID profile? The stakeholders of the PTCRIS could be the national government or the university of the researcher who wants all publications listed of a researcher (i.e. the numbers counts in assessments) whereas the researcher may only want a selected number of publications (e.g. the best) to be listed visible. This is briefly discussed in the conclusion. It could be suggested to separate discussion and future perspectives i.e. to a separate section.
3. The metadata quality must be a major challenge to this synchronization framework using ORCID. It is well-known that metadata about research outputs and funding data are often not consistent or of an acceptable quality. It is not clear from the article how the CRIS infrastructure is used in Portugal. If it is used for a research assessment i.e. bibliometric assessment this must be a major issue. It would be relevant to have discussed this issue a bit further in the paper. However since the paper is presenting a prototype framework – it is acceptable that it is not dealt with in details. I hope that this will be explored further in later writings from the authors – also when ORCIDs API version 2.0 will be in production and the metadata roundtrip also will be in production.
4. Annotations – there are some use of annotations like SYNCED (o, p) where *o, p *could have been described to help the reader.
5. The description and use of PTCRIS sometimes gives the impression of PTCRIS as a single entity or system – while as far as this reviewer understands it is an umbrella that encompasses several systems that together collects and exposes the collected research output, actors and activities of Portugal. It currently includes: DeGóis, RCAAP and SARI.
6. The authors do not discuss issues with researchers having more ORCIDs – is this not an issues for the proposed synchronization framework?

Overall the paper is solid and provides an important stepping stone towards a better use and re-use of research information using ORCID as a hub. And I would be happy to accept the paper as is – but I hope that the comments will provide thought for possible revisions or further work in a later article.

I have read this submission. I believe that I have an appropriate level of expertise to confirm that it is of an acceptable scientific standard.
